# Taxonomic Organization of the Family *Brucellaceae* Based on a Phylogenomic Approach

**DOI:** 10.3389/fmicb.2019.03083

**Published:** 2020-01-30

**Authors:** Sébastien O. Leclercq, Axel Cloeckaert, Michel S. Zygmunt

**Affiliations:** INRA, Infectiologie et Santé Publique, Université de Tours, Nouzilly, France

**Keywords:** *Brucella*, *Ochrobactrum*, *Rhizobiales*, *Mycoplana*, phylogenomic reconstruction

## Abstract

Deciphering the evolutionary history of pathogenic bacteria and their near neighbors may help to understand the genetic or ecological bases which led to their pathogenic behavior. The *Brucellaceae* family comprises zoonotic pathogenic species belonging to the genus *Brucella* as well as the environmental genus *Ochrobactrum* for which some species are considered as opportunistic pathogens. Here, we used a phylogenomic approach including a set of 145 *Brucellaceae* genomes representative of the family diversity and more than 40 genomes of the order *Rhizobiales* to infer the taxonomic relationships between the family’s species. Our results clarified some unresolved phylogenetic ambiguities, conducting to the exclusion of *Mycoplana* spp. out of the family *Brucellaceae* and the positioning of all *Brucella* spp. as a single genomic species within the current *Ochrobactrum* species diversity. Additional analyses also revealed that *Ochrobactrum* spp. separate into two clades, one comprising mostly environmental species while the other one includes the species considered as pathogens (*Brucella* spp.) or opportunistic pathogens (mainly *O. anthropi*, *O. intermedium*, and *O. pseudintermedium*). Finally, we show that *O. intermedium* is undergoing a beginning of genome reduction suggestive of an ongoing ecological niche specialization, and that some lineages of *O. intermedium* and *O. anthropi* may shift toward an adaption to the human host.

## Introduction

The *Brucellaceae* is a family of Gram negative bacteria, member of the order *Rhizobiales* within the class *Alphaproteobacteria*. The family was named after the genus *Brucella*, a facultative intracellular pathogen responsible for the zoonotic disease brucellosis, which causes major economical burden in livestock and human health concerns worldwide (Ficht, 2010; Garin-Bastuji et al., 2014). First described *Brucella* species were specifically associated with livestock animals, such as sheep and goats for *B. melitensis*, cattle for *B. abortus*, and pigs for *B. suis*, in which they can cause abortions and other reproductive diseases. These species are highly transmissible to humans through direct contacts with infected animals, aerosols, or consumption of raw-milk dairy products, and can produce chronic, debilitating infections. Other pathogenic *Brucella* species were later described, such as *B. canis* causing infection in dogs and which is also pathogenic in humans, and *B. ovis* causing epididymitis in rams (Buddle, 1956; Carmichael and Bruner, 1968). Other species have been isolated from wildlife and consist in their order of description of (i) *B. neotomae* isolated from rodents (Stoenner and Lackman, 1957), (ii) *B. ceti* and *B. pinnipedialis* isolated from marine mammals (Foster et al., 2007), (iii) *B. microti* initially isolated from the common vole but later found in a wider variety of animals such as red foxes, wild boar and even frogs (Jay et al., 2018; Scholz et al., 2008b), (iv) *B. papionis* isolated from baboons (Whatmore et al., 2014), and (v) *B. vulpis* isolated from red foxes (Scholz et al., 2016). More genetically distant *Brucella* strains comprising several potential new species, have been also reported (Tiller et al., 2010; Eisenberg et al., 2012, 2016). To date only one species of this group of strains has validly been published as *B. inopinata*, and consists of a single human isolate for which the animal or environmental reservoir has not yet been identified (De et al., 2008; Scholz et al., 2010). The pathogenic status for humans of the newly described wildlife or environmental species is currently not well understood although some human clinical isolates with a similar genetic background have been reported (Sohn et al., 2003; McDonald et al., 2006; Scholz et al., 2010; Tiller et al., 2010; Cloeckaert et al., 2011). One of the striking specificity of the *Brucella* genus is its extreme homogeneity at the genetic level, with more than 90% DNA-DNA hybridization between species (Verger et al., 1985). Despite this lack of diversity, the phylogenetic relationship between species was extensively investigated using various genetic tools and more recently with the availability of whole-genome sequences, and are now well resolved (Foster et al., 2009; Wattam et al., 2014, Al Dahouk et al., 2017). The current classification includes 12 *Brucella* species commonly classified as ‘classical’ or ‘atypical’, depending on their phenotypical characteristics and their phylogenetic relationships (Scholz et al., 2018).

The other well-studied genus in the family *Brucellaceae* is *Ochrobactrum*. There are currently 18 *Ochrobactrum* species described, with the recent re-classification of *O. lupini* as an *O. anthropi* synonym (Gazolla Volpiano et al., 2019), which are mostly considered as environmental bacteria. For example, *O. tritici*, *O. oryzae*, and *O. cystisi* were first isolated from plant rhizosphere (Lebuhn et al., 2000; Tripathi et al., 2006; Zurdo-Pineiro et al., 2007), *O. anthropi*, *O. grignonense*, *O. intermedium* can be isolated from bulk soil (Lebuhn et al., 2000; Scholz et al., 2008a), while the type strain of *O. thiophenivorans* was isolated from wastewater (Kämpfer et al., 2007). Nonetheless, some few species, such as *O. anthropi*, *O. intermedium*, *O. pseudintermedium, O. haematophilum*, were initially isolated from human clinical cases (Holmes et al., 1988; Velasco et al., 1998; Kämpfer et al., 2007, 2008; Teyssier et al., 2007) and among them the two first seem to be the main species involved in opportunistic human infections (Teyssier et al., 2005). The other members of the family *Brucellaceae* family are the more distant *Pseudochrobactrum*, *Paenochrobactrum*, *Falsochrobactrum, Mycoplana* and *Daeguia* genera, composed of four, three, two, two, and one species, respectively (Yoon et al., 2008; Kämpfer et al., 2014; Kämpfer and Glaeser, 2015; Urakami and Segers, 2015; Sun et al., 2019). Although *Paenochrobactrum*, *Pseudochrobactrum*, and *Falsochrobactrum* genera seem to be true *Brucellaceae*, the inclusion of the two latter genera in the family is still a matter of debate (Kämpfer and Glaeser, 2015). The phylogenetic relationships between *Brucellaceae* species have been much less thoroughly investigated than those belonging strictly to the genus *Brucella*, and most studies relied on the comparison of the small subunit 16S rRNA or the *recA* gene. The most comprehensive analysis of *Brucellaceae* species to date, that included more than one hundred strains, showed that the use of these single markers results in different tree topologies depending on the locus and the phylogenetic reconstruction algorithm, and with low to very low branching support throughout the topology (Scholz et al., 2008a). Such lack of resolution was confirmed by a number of other studies which each reported a unique tree topology using the same markers (Teyssier et al., 2007; Huber et al., 2009; Kämpfer et al., 2013, 2015; Chai et al., 2015; Gazolla Volpiano et al., 2019; Krzyżanowska et al., 2019). Nonetheless, a striking common observation of these various studies is that the genus *Brucella* was consistently embedded within the *Ochrobactrum* genus diversity, although no definitive conclusion about the taxonomic status of *Brucella* spp. could be drawn because of the underlying lack of phylogenetic resolution. Limited multilocus sequence typing (MLST) or Internal Transcribed Spacer (ITS) region-based analyses were also performed but were hampered by a small set of genes, a small set of species, or poorly supported branching, and still resulted in conflicting results (Zurdo-Pineiro et al., 2007; Aujoulat et al., 2014; Burygin et al., 2019; Gazolla Volpiano et al., 2019; Krzyżanowska et al., 2019). More recently, whole genome phylogenetic studies started to emerge but generally focused on a limited number of isolates of few species and could not provide a comprehensive view of the phylogenetic relationships at the family level or even at the *Ochrobactrum* genus level (Gazolla Volpiano et al., 2019; Krzyżanowska et al., 2019). Finally, the question of the *Brucellaceae* positioning among the *Rhizobiales* order have not been thoroughly investigated to date. Depending on the studies, the *Brucellaceae* family is a sister group of the *Bartonellaceae*, the *Rhizobiaceae*, or the *Phyllobacteriaceae* (Gupta and Mok, 2007; Scholz et al., 2008a; Kämpfer et al., 2015). In the present study, the taxonomic organization of the *Brucellaceae* genera and their species among the *Rhizobiales* order was determined at the genomic scale using a representative sample of genome sequences available in GenBank, and the inter-relationship between members of the family *Brucellaceae* was investigated using a comprehensive phylogenomic analysis including most GenBank genomes and newly sequenced isolates.

## Materials and Methods

### Genome Sequences

The 101 non-*Brucella Brucellaceae* genomes available in the GenBank assembly summary file as of 07/14/2019 were downloaded from the NCBI FTP repository^1^ in fasta format. Since almost 800 *Brucella* genomes were available at the time of this study and that the phylogenomic organization of the genus was already thoroughly investigated before (Wattam et al., 2014; Al Dahouk et al., 2017), only 12 genomes spanning the diversity of the classical and non-classical *Brucella* species were used in our analyses (Supplementary Table S1). A set of 43 diverse non-*Brucellaceae Rhizobiales* genomes listed as ‘reference’ or ‘representative’ genomes in the NCBI database were also downloaded, as well as the genome of two *Caulobacteriales* (*Caulobacter vibrioides* and *Parvularcula bermudensis*) to serve as outgroups. GenBank assembly identifiers of all downloaded genomes are given in Supplementary Table S1. Additionally, the genomes of 32 *Ochrobactrum* spp. isolates were sequenced and added to the analysis. For the 32 isolates, bacterial DNA was extracted with DNeasy Blood & Tissue kit from QIAGEN. Genome sequencing was performed by Genoscreen (Lille, France) on an Illumina MiSeq apparatus (paired end 250 bp), with an average of 60x coverage per genome, except for the *Ochrobactrum* sp. strain Kaboul genome which was sequenced on a Pacific Bioscience SMRT device. Resulting reads were quality trimmed with Trimmomatic v.0.33 (Bolger et al., 2014) and assembled using the Spades v.3.11.1 assembly software (Bankevich et al., 2012). Information and accession/assembly number of these newly sequenced genomes are given in Supplementary Table S2.

### Phylogenomic Analyses

All genomic nucleotide sequences were first annotated with PROKKA 1.12 (Seemann, 2014) using the same parameters, i.e., default parameters with no search of ribosomal and transfer RNA, to ensure consistent ORF detection among all sequences. At this stage, the genome sequence of *O. anthropi* SUBG007 was excluded from further analysis because of an excessive number of very short predicted proteins, suggesting a poor assembly quality. The remaining annotated proteomes were then used to create two datasets. Dataset 1 included all non-*Brucellaceae Rhizobiales* proteomes, the two *Caulobacteriales* proteomes, and 25 *Brucellaceae* proteomes representative of the family diversity. Dataset 2 included all downloaded and locally sequenced *Brucellaceae* proteomes as well as four other *Rhizobiales* proteomes to serve as outgroups. Proteomes of *Bartonella* species were not selected as outgroups despite being the closest *Brucellaceae*’s family because their highly reduced genomes would lower the number of proteins shared with the *Brucellaceae* and reduce the power of the analysis. Protein orthology relationships were inferred for both datasets independently using the OrthoMCL pipeline (Li et al., 2003) based on all-against-all BlastP hits with an *e*-value of 10^–20^. Among the 20,319 and 16,717 orthology groups returned, respectively 145 and 195 were retained because they included only orthologs (detected only once in each proteome) and were present in 100% and at least 95% of all proteomes, including all outgroups. We used 95% ortholog prevalence threshold for the *Brucellaceae* analysis because this dataset included a number of genomes originating from metagenomic data (Supplementary Table S1) for which some conserved genomic regions were missing. Proteins of each orthogroup were aligned independently using MAFFT v.7.310 (Katoh and Standley, 2013) with the L-INS-I method and the resulting alignments were concatenated in a single large alignment for each dataset, of 78512 and 79,793 residues respectively, in which missing proteins in some orthogroups were replaced by tracks of gaps. To select the model of protein evolution that best fitted our two datasets, we performed a PROTTEST 3.4.2 preanalysis (Darriba et al., 2011). The program returned the LG model (Le and Gascuel, 2008) with a gamma model of rate heterogeneity, a proportion of invariable sites, and an empirical residue frequency as the best model for both datasets. RAxML v8.2.11 (Stamatakis, 2006) was used to compute a maximum likelihood tree for each concatenated alignment, using the rapid bootstrap algorithm with the protein model returned by the PROTTEST analysis (GAMMAILGF) and 100 bootstraps. Additional phylogenomic reconstructions were computed to test the positioning of *Falsochrobactrum* spp. genomes within the resulting trees. Each reconstruction was performed on the same selection of 25 *Brucellaceae* genomes used in Dataset 1 (and the genome of *Mesorhizobium loti* as an outgroup), for which *F. ovis* genome, *F. shanghaiense* genome, or none were discarded, respectively. Trees were calculated for these three combinations using each of the 145 and 195 orthogroups alignments obtained from Dataset 1 (*Rhizobiales* analysis) and Dataset 2 (*Brucellaceae* analysis), with RaxML and the same parameters as in the two first calculations. All trees were displayed and annotated using the iTOL v.4 web interface (Letunic and Bork, 2019).

### 16S rRNA Sequence Alignment Analysis

The 16S rRNA sequence was extracted from each *Brucellaceae* genome used in Dataset 1 and were completed with 15 16S rRNA sequences from type strains of all *Brucellaceae* species with no genome available, downloaded from NCBI. The sequence of some other *Rhizobiales* were also added for comparison purposes. Pooled fasta were aligned using MUSCLE (Edgar, 2004) with default parameters using Ugene v.1.30 (Okonechnikov et al., 2012). The alignment was manually inspected and unexpectedly similar sequence stretches found in phylogenetically distant strains were considered as hallmarks of past recombination events.

### gANI Analysis

All *Brucellaceae* annotated genomes from Dataset 2 were subjected to a pairwise gANI calculation using ANIcalculator v.1 (Varghese et al., 2015) with rRNA genes excluded. As recommended in Varghese et al. (2015), all genome pairs with a gANI value > 96.5 were considered belonging to the same species.

### Core and Pan-Proteome Analysis

Core and pan proteomes were computed independently on *Brucellaceae* phylogenetic clades including at least five isolates and corresponding to species according to the gANI criterion. Five genomes of *O. intermedium*, 10 genomes of *O. anthropi* and 4 genomes of *O. pseudogrignonense* clades were excluded from the analysis because they were reconstructed from metagenomic sequencing and lacked large core-genome regions, which resulted in inconsistent results. Additionally, no contig of strain BH3 (*O. pituitosum* clade) matched the second chromosome of *Ochrobactrum*, suggesting incomplete sequencing. This genome was therefore also excluded from the analysis. Calculations were performed using a in-house procedure (detailed below) and were then confirmed using the Roary software (Page et al., 2015) with a 95% blastp minimum identity, and genes defined as core when present in 100% of isolates. The in-house procedure started with a within-species all-vs.-all blastp comparison with an *e*-value of 10^–20^, at least 80% coverage of both sequences, and a post-treatment filtering to keep only hits with ≥95% amino-acid identity. All filtered result files were parsed to group proteins into homology clusters, by recursively combining hits matching to the same proteins. For each homology group, the number of core and pan protein-coding genes were defined as the minimum and maximum number of homologues in a single genome, respectively. Core and pan numbers were summed over all homology groups to obtain the total core and pan genomes. Both calculations (in-house and Roary) returned core and pan genomes of each genomic species. Because each species has a different number of sequenced genomes, accumulation curves for the core and accessory (pan minus core) genomes were calculated as follows: for each *x* between 1 and *N*, with *N* the total number of genomes in the group, 50 random combinations of *x* genomes among *N* were selected, and their core and accessory genomes were estimated.

## Results and Discussion

### Phylogenomic Positioning of the Family *Brucellaceae* in the Order *Rhizobiales*

**FIGURE 1:**
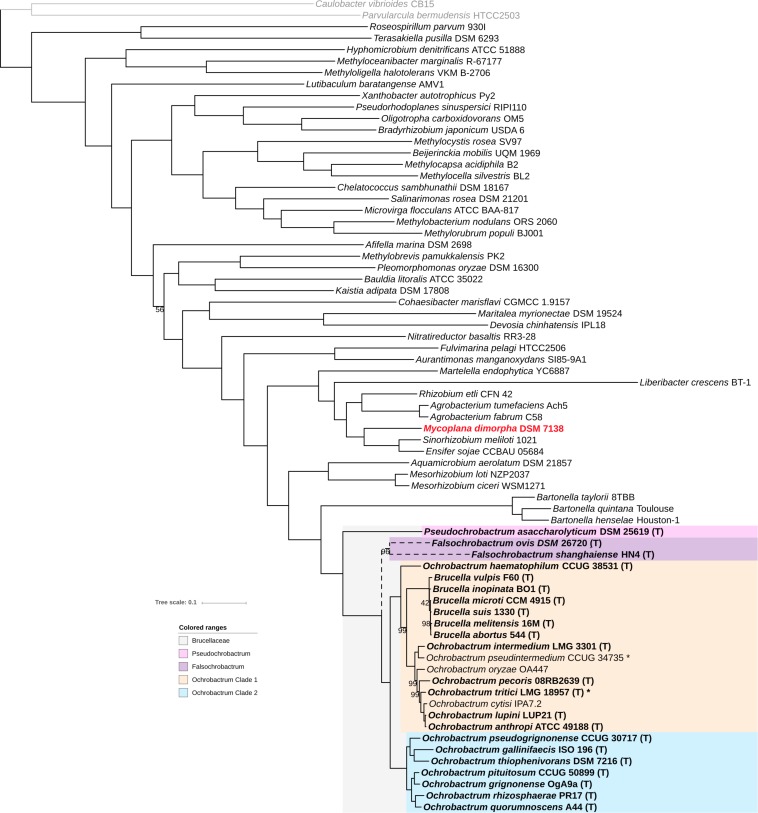
Position of the *Brucellaceae* genomes in the *Rhizobiales* phylogeny. The ML phylogenetic tree was computed using RaxML from an alignment of 145 core proteins, and rooted using two *Caulobacteriales* genomes (depicted in gray). Branching support was estimated using 100 bootstrap replicates, with only values lower than 100% displayed. The *Brucellaceae* clade is indicated with gray background. *Brucellaceae* type strains are highlighted with bold labeling. A star indicates a genome sequenced in the current study. *Pseudochrobactrum*, *Falsochrobactrum*, and *Ochrobactrum* clade 1 and clade 2 are indicated with pink, purple, orange, and blue backgrounds, respectively. The misclassified *Mycoplana dimorpha* genome is labeled in red. Branching of *Falsochrobactrum* spp. is depicted in dashed lines to reflect conflicting topology with the phylogeny displayed in Figure 2.

Forty-three complete and draft genomes representative of the *Rhizobiales* diversity and 25 genomes representative of the *Brucellaceae* diversity from the NCBI ftp repository (Supplementary Table S1) were selected, and a phylogenomic tree from a set of 145 proteins shared by all genomes (core proteins) was constructed. As seen in Figure 1, the high number of compared proteins resulted in an almost completely resolved tree, with all but one bootstrap values being ≥98%. The tree structure generally reflects the currently accepted *Rhizobiales* families grouping, with few exceptions. Consistent with 16S rRNA and *recA* analyses, *Pseudochrobactrum* and *Falsochrobactrum* spp. form a monophyletic group with other *Ochrobactrum* and *Brucella* spp. On the contrary, the *Mycoplana dimorpha* genome investigated here branches with maximum bootstrap support distant to the *Brucellaceae* family, and actually within the family *Rhizobiaceae* (Figure 1). *Mycoplana stricto sensu* have been variously included into the *Mycobacteriaceae*, the *Pseudomonadaceae*, or even left unclassified before ending up into the *Brucellaceae* (Urakami and Segers, 2015). However, this classification was not based on phylogenetic studies, and most current phylogenies actually branches the genus outside the *Brucellaceae* (Yanagi and Yamasato, 1993; Scholz et al., 2008a; Kämpfer and Glaeser, 2015). This results provide the first robust taxonomic position for *Mycoplana*, which indicates that the genus should be transferred from the *Brucellaceae* to the *Rhizobiaceae*. Our fully resolved tree also indicates that the sister group of the *Brucellaceae* family is the *Bartonellaceae* family with 100% confidence. This result is consistent with another phylogenomic study that spanned the whole α-Proteobacteria diversity and therefore included only a few *Brucella* species as representative of the *Brucellaceae* (Gupta and Mok, 2007). Finally, the extensive *Rhizobiales* genome diversity used here provide a clear delineation of the *Brucellaceae* family and confirms that no other *Rhizobiales* species/genus are part of the family.

### Phylogenomic Relationships Among *Brucellaceae*

Our next objective was to better understand the intra-family phylogenetic relationships between *Brucellaceae* species. In the topology observed in Figure 1, *Pseudochrobactrum asaccharolyticum* branches at the root of the *Brucellaceae*, and *Falsochrobactrum* spp. branch as a sister clade of all *Ochrobactrum* and *Brucella* species. All these other species are then separated into two main clades. The first clade includes *Ochrobactrum* species *haematophilum*, *pseudintermedium, intermedium*, *oryzae, pecoris, tritici, cytisi*, and *anthropi-lupini*. The triad *anthropi-lupini*/*cytisi*/*tritici* was generally well resolved in most previous phylogenies, but the position of *O. oryzae, O. pecoris*, and *O. haematophilum* was much less consistent. 16S rRNA-based analyses usually branch *O. oryzae* with *O. pseudintermedium* and *O. gallinifaecis*, away from the *anthropi*/*tritici* clade, although *recA* analyses provided results in agreement with our phylogenomic analysis (Scholz et al., 2008a; Huber et al., 2009; Gazolla Volpiano et al., 2019; Krzyżanowska et al., 2019). Similarly, *O. pecoris* usually branches within or as a sister taxon of *Ochrobactrum* Clade 2 (see below) in 16S rRNA-based phylogenies, with sometimes a close connection to *O. rhizospherae* and *O. pituitosum* (Kämpfer et al., 2011, 2013, 2015; Krzyżanowska et al., 2019), while the MLST phylogeny more accurately branched it with the other members of *Ochrobactrum* Clade 1 (Aujoulat et al., 2014). For *O. haematophilum*, no single-locus or even multi-locus analysis could have resolved its correct position with high support (Scholz et al., 2008a; Aujoulat et al., 2014; Gazolla Volpiano et al., 2019), suggesting that its deep branching at the root of Clade 1 precludes from retrieving enough phylogenetic signal when only few genes are used. It should also be noted that all *Brucella* species form a monophyletic cluster inside Clade 1 with maximum support. The inclusion of *Brucella* among *Ochrobactrum* species was recurrently observed within previous studies, but the exact positioning have never been conclusively resolved (Zurdo-Pineiro et al., 2007; Huber et al., 2009; Kämpfer et al., 2015; Burygin et al., 2019). Specifically, the species *O. intermedium* was named after its supposed closest relationship to *Brucella* spp. using serological cross-reactivity, polymyxin resistance, and 16S rRNA-based phylogeny (Velasco et al., 1998), a clustering also observed in most 16S rRNA-based analyses (Kämpfer et al., 2007, 2008; Teyssier et al., 2007; Zurdo-Pineiro et al., 2007; Scholz et al., 2008a; Huber et al., 2009). Here we show that *Brucella* spp. branch as a sister group of a clade including the *Ochrobactrum* species *pseudintermedium*, *intermedium*, *oryzae*, *perocis*, *cytisi*, *tritici*, and *anthropi-lupini.* It indicates that *O. intermedium* is actually more genetically related to most *Ochrobactrum* spp. of Clade 1 than to *Brucella* spp.

Clade 2 includes *Ochrobactrum* species *gallinifaecis*, *thiophenivorans*, *pseudorignonense*, *quorumnoscens*, *rhizospherae*, *grignonense*, and *pituitosum*. These species consistently group together in most 16S rRNA-based phylogenetic analyses, but usually as an internal *Ochrobactrum* clade and with *O. gallinifaecis* excluded (Chai et al., 2015; Gazolla Volpiano et al., 2019; Krzyżanowska et al., 2019). On the contrary, *recA* and multilocus phylogenies usually separated correctly this group from the other *Ochrobactrum spp.* (Aujoulat et al., 2014; Kämpfer et al., 2015; Gazolla Volpiano et al., 2019), suggesting that some recombination events in the 16S rRNA region have blurred the phylogenetic signal for this clade.

### Comparison of the 16S rRNA Region

The numerous discrepancies revealed in the previous section between our phylogenomic analysis and 16S rRNA-based phylogenies prompted us to investigate in more detail the 16S rRNA sequence of the *Brucellaceae.* We constructed an alignment using the 16S rRNA sequence extracted from all *Brucellaceae* genomes and several outgroups from Figure 1, completed with 16S rRNA sequences for the *Brucellaceae* type strains not available as genome sequences in the NCBI database. The resulting alignment show numerous likely recombined regions involving diverse *Ochrobactrum* and non-*Ochrobactrum* species (see Table 1 and Supplementary Figure S1). For instance, the *O. gallinifaecis* ISO 196 16S rRNA sequence is highly recombined, with three independent inferred recombined regions, all three of them shared with non-*Ochrobactrum* species or possibly with *Aquamicrobium aerolatum* from the family *Phyllobacteriaceae* (Table 1). These observed recombination events are likely the reason for the incorrect positioning of *O. gallinifaecis* outside *Ochrobactrum* clade 2 in previous 16S rRNA-based phylogenies. Interestingly, one of them is shared with *O. pseudintermedium* and two with *O. oryzae*, two species usually grouped along with *O. gallinifaecis* in 16S rRNA phylogenies. The grouping of *O. pecoris* with *Ochrobactrum* clade 2 in 16S rRNA phylogenies is also probably caused by the recombination event inferred at positions 75–91 of the alignment, which also involves *O. rhizospherae*, *O. pituitosum*, and *O. daejeonense* (Table 1). Another recombined region is inferred at pos. 927–979 in the alignment and involves the whole clade including *O. tritici, O. cytisi*, and *O. anthropi-lupini with Ochrobactrum* Clade 2 (Table 1). Since this region is one of the highly variable region of the 16S rRNA sequence (see Supplementary Figure S1), the resulting inter-species similarity likely weighted on the positioning of *Ochrobactrum* Clade 2 within *Ochrobactrum* Clade 1 in former studies. Likewise, it forces the *O. anthropi-tritici* clade to branch out of *Ochrobactrum* Clade 1, leaving *O. intermedium* as an apparent sister clade of the *Brucella* spp. in these studies. Finally, a number of other discrepancies were noticed in the 16S rRNA alignment (green boxes in Supplementary Figure S1), which contributes to the general unreliability of phylogenies based on the 16S rRNA sequence. In contrast, no obvious recombined region involving specifically *Mycoplana* and any member of the *Brucellaceae* was observed, consistent with its highly supported positioning among the family *Rhizobiaceae*.

**TABLE 1:** Inferred recombination regions in 16S rRNA sequence of *Brucellaceae* isolates.

	*O.oryzae*	*O.pseudintermedium*	*O.quorumnoscens*	*O.rhizosphaerae O. pituitosum O. daejeonense*	*Ochrobactrum* Clade 2	*Paenochrobactrum* spp.	*Pseudochrobactrum* spp. *Falsochrobactrum* spp.	*D.caeni*	*A.aerolatum*
*O.gallinifaecis* ISO 196 (T)	63–178*	63–178				63–178		371–378	
	519–577		371–378			519–577	519–577		519–577
*O.pecoris* 08RB2639 (T)				75–91		75–91			
*O.anthropi-tritici* clade					927–979				

*Numbers refer to positions in the 16S rRNA alignment provided in Supplementary Figure S1.

### *Falsochrobactrum* Species Phylogenetic Positioning

**FIGURE 2:**
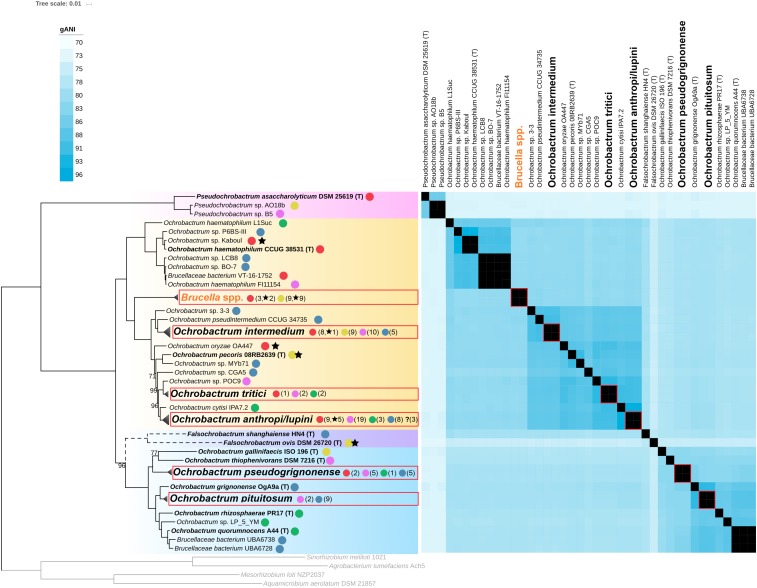
ML Phylogenetic tree of the *Brucellaceae* clade. The tree was computed using RaxML from an alignment of 195 core proteins, and rooted using four members of the *Rhizobiaceae* and *Phyllobacteriaceae* families (depicted in gray). Branching support was estimated using 100 bootstrap replicates, with only values lower than 100% displayed. Genomes of type strains are in bold font. The *Pseudochrobactrum* clade is indicated with pink background, the *Falsochrobactrum* in purple background, and the *Ochrobactrum* Clades 1 and 2 are indicated with orange and blue backgrounds, respectively. Monophyletic clades corresponding to genomic species and including 5 genomes or more are collapsed and boxed in red. Branching of *Falsochrobactrum* spp. is depicted in dashed lines to reflect conflicting topology with the phylogeny displayed in Figure 1. Origin of strain isolation is indicated with a colored dot: red, human; yellow, animal; pink, anthropized environment; green, plant rhizosphere; blue, other (environmental). Numbers in parenthesis indicate the number of genomes of each origin for collapsed clades. Stars indicate human or animal clinical isolates. The heatmap represent pairwise genome gANI values from 70 (light blue) to 96.5 (deep blue). Values larger than 96.5 (the genomic species threshold) are displayed in black. Collapsed gANI values are boxed in red.

To go further in the taxonomic analysis of the *Brucellaceae*, a new core-proteins tree was constructed, including all non-*Brucella Brucellaceae* genomes available in GenBank at the time of the study (15th of July, 2019), 12 *Brucella* genomes representative of the diversity of the genus, and 32 novel *Ochrobactrum* genomes sequenced in the present study (see Supplementary Tables S1, S2). The topology of this new tree is highly congruent with the topology observed for the *Brucelleaceae* type strains in the *Rhizobiales* tree, except for the position of *Falsochrobactrum* spp. (Figure 2 and Supplementary Figure S2). Indeed, although the two *Falsochrobactrum* species cluster together in both topologies, they branch with maximum support as a distinct genus in the *Rhizobiales* tree (Figure 1) but within the *Ochrobactrum* diversity in the *Brucellaceae* tree (Figure 2). To test whether this discrepancy was caused by the set of included genomes or by the set of core proteins used, additional phylogenetic trees were constructed using the same representative *Brucellaceae* type strains with/without *Falsochrobactrum* genomes, and with each core protein dataset. When the two *Falsochrobactrum* genomes are included in the analysis, both calculations provide the same topology than the original trees (see Supplementary Figures S3A,B). When each single *Falsochrobactrum* genome is included alone, the branching is similar whatever the protein dataset: outside the *Ochrobactrum* clade for *F. ovis*, and inside the *Ochrobactrum* clade for *F. shanghaiense* (see Supplementary Figures S3C–F). This inconsistent positioning suggests that extensive recombination events occurred in *F. shanghaiense* or *F. ovis*, or both, and questions the classification of *Falsochrobactrum* spp. as a new genus, at least from a genomic point of view. Unfortunately, the deep branching of these genomes within the *Brucellaceae* and the lack of sequenced close relatives hamper any further investigation using existing algorithm of recombination detection. The *Falsochrobactrum* spp. phylogenetic position in the family *Brucellaceae* thus cannot be accurately determined in the present study, and its resolution will require additional sequencing effort.

### Delimitation of *Ochrobactrum* Genomic Species

The high resolution of our *Brucellaceae* core protein phylogeny emphasized the presence of clusters that generally overlap with the species names given to isolates (see Supplementary Figure S2). To delineate even more precisely species boundaries at the genomic level, an all-versus-all gANI comparison was conducted on the *Brucellaceae* genomes. A gANI score is a measure of genomic relatedness which can be viewed as an *in silico* DNA-DNA hybridization (Goris et al., 2007), and benchmarking on more than 13,000 genomes representative of most of the accepted bacterial species indicated that a genome pair with a gANI value > 96.5 can be considered belonging to the same species (Varghese et al., 2015). In the following sections, cluster of genomes with a gANI higher than this threshold are named genomic species, to avoid any ambiguity with taxonomically accepted species. In our phylogenomic framework, most clusters with genomes harboring the same species name match a single genomic species, supporting the robustness of the gANI indicator to detect species boundaries. The *O. haematophilum* cluster is an exception, with two genomes (*O. haematophilum* L1Suc and *O. haematophilum* FI11154) distantly related to the type strain genome, with 86.37 and 90.21 gANI values, respectively (Figure 2). The strain FI11154 was identified as *O. haematophilum* solely on the closest relationship of its 16S rRNA sequence to the *O. haematophilum* type strain (Diaz et al., 2018) while no information about the characterization process is available for strain L1Suc. It is thus likely that these two strains do not belong to the species *O. haematophilum* and should return to an unidentified *Ochrobactrum* sp. status until more thorough characterization. The gANI analysis also indicated that *O. cytisi* IPA7.2 can be clearly separated from *O. anthropi* genomes (gANI in the range 95.47–95.99) despite a very close phylogenetic relationship (Figure 2). Although the sequenced strain is not the type strain of the species, a multilocus analysis showed previously that IPA7.2 is phylogenetically closer to the *O. cytisi* type strain than to any *O. anthropi* strain (Burygin et al., 2019). These results support the status of *O. cytisi* as a separate species, a status which have been recently questioned based on 16S rRNA, MLSA and DDH analyses (Gazolla Volpiano et al., 2019). On the contrary, gANI calculation indicates that all *Brucella* genomes included in this study, which span the whole diversity of currently known *Brucella* species, actually belong to the same genomic species. This result is fully consistent with previous DNA-DNA hybridization and 16S rRNA analyses and support the concept that all currently known members of the genus *Brucella* constitute in fact a single species, at least from a genetic point of view (Verger et al., 1985). The genomic species delineation finally confirmed that *O. lupini* isolates fall in the diversity of the *O. anthropi* species (Gazolla Volpiano et al., 2019) and revealed that a number of genome sequences available in GenBank were incorrectly identified. Indeed, phylogenomic and gANI analyses indicate that *O. anthropi* FRAF13 belongs to the *tritici* clade, *O. pituitosum* AA2 to the *pseudogrignonense* clade, and both *O. rhizospherae* SJY1 and *O. thiophenivorans* MYb6 to the *pituitosum* clade (see Supplementary Figure S2).

### Core and Accessory Genomes Among *Ochrobactrum* Species

**FIGURE 3:**
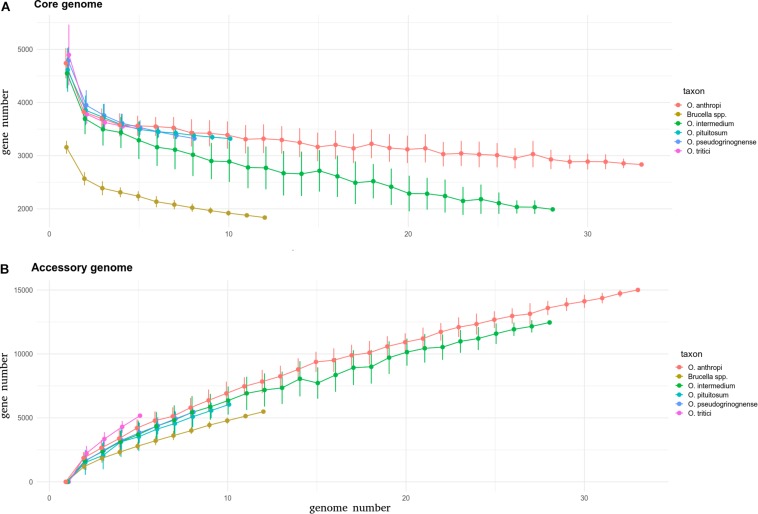
Core **(A)** and accessory **(B)** genomes of *Brucella* and various *Ochrobactrum* species calculated using an in-house pipeline. For each species, the graph shows the number of genes in core and accessory genomes estimated for an increasing number of considered genomes, up to the number of genomes available for the taxon. The core genome is defined as the set of genes detected in all genomes under consideration. The accessory genome is defined as the total of distinct genes detected in the considered genomes (pan genome) minus the core genome. Since not all taxa have the same number of sequenced genomes, accumulation curves were produced as follows: the number of considered genomes increases from one to the total number of genomes in the taxon, and at each step, the considered genomes are selected randomly in the pool of genomes available for the taxon. This random selection was repeated up to 50 times at each step and median and standard error were estimated for core and accessory gene numbers (see section Materials and Methods).

After considering these species boundaries at the genomic level, five *Ochrobactrum* species have more than four sequenced genomes, with a diversity suitable for core and pan genome estimations. Among them, *O. anthropi* and *O. intermedium* were the most heavily sequenced, with 43 and 33 genomes available, respectively. *O. pituitosum*, *O. pseudogrignonense*, and *O. tritici* followed, with 11, 8, and 5 genomes available, respectively. All these species have ∼4300–5000 protein-coding genes, with no significant difference between species (Figure 3A, ANOVA test, *p* = 0.061). By comparison, the genomes of the 12 *Brucella* isolates, known to have experienced genomic reduction (Wattam et al., 2014), harbor ∼3200 protein-coding genes on average. When the core genome of each species is estimated, differences emerge. While the species *anthropi*, *pituitosum*, *pseudogrignonense*, and *tritici* species show a similar decreasing curve with an increasing number of observed genomes, *O. intermedium* exhibits a faster and stronger decrease (Figure 3A). When 28 genomes are included in the comparison (the maximum possible for *O. intermedium* after discarding incompletely sequenced genomes), the core genome of *O. intermedium* is almost 1000 genes smaller than those of *O. anthropi*, a result supported by two independent calculation methods (see Section Materials and Methods, Supplementary Figure S4A). Such difference is not compensated by a larger accessory genome (all non-core protein-coding genes) for which all *Ochrobactrum* species except *O. tritici* show a similar increase in the accessory genome with increasing number of genomes observed (Figure 3B and see Supplementary Figure S4B). Overall, these results suggest that *O. intermedium* could be subject of pervasive gene disruption, with no or very few gene inactivation yet fixed in the population. Genome-wide gene disruption is considered as one of the first steps of genome reduction, usually caused by the reduction of effective population size following ecological niche specialization, such as host-adaptation (Toft and Andersson, 2010). Consistent with our observation, an ongoing process of genome reduction in *O. intermedium* have already been proposed, based on the recurrent deletion of large genomic regions in different strains (Aujoulat et al., 2014). The authors of this study compared the population structure and isolation site of 65 *O. intermedium* isolates and concluded that the species may undergo a niche specialization toward an environment related to medical and industrial technologies. Another study suggested that *O. intermedium* may be specializing to the animal gastric niche, based on co-isolation and genetic features shared with the stomach-adapted human pathogen *Helicobacter pylori* (Kulkarni et al., 2014). The large number of human-related *O. intermedium* isolates (Scholz et al., 2008a; Aujoulat et al., 2014), sometimes from human clinical cases (Velasco et al., 1998; Moller et al., 1999), as well as their intrinsic resistance to polymyxin E (Velasco et al., 1998), a trait shared with *Brucella* spp., also suggest that *O. intermedium* may shift to an animal-associated and potential pathogenic lifestyle.

### Ecological Niches of *Brucellaceae* Species

The intriguing observation made for *O. intermedium* genomes suggests that isolation sources for the sequenced strains may inform us on its potential niche specialization. We therefore compiled this information for all the *Brucellaceae* and classified them into five main sources: human clinical, human-related environments, vertebrate animals (including strains causing diseases), plant rhizosphere, and natural environment (including invertebrate animals such as worms). The distribution of these main sources among the *Brucellaceae* tree revealed a clear separation between *Ochrobactrum* Clade 1 and Clade 2, with almost all human, human-related, and animal isolates being localized in Clade 1 while Clade 2 comprises mainly environmental and plant-associated isolates (Figure 2). Strikingly, Clade 1 is actually those in which the *Brucella* spp. belong to, suggesting that this taxonomic lineage may harbor genetic determinants facilitating a preferential association with animal hosts, including humans. At the species level, *O. intermedium* shows the lowest proportion of environmental isolates and many isolates sampled in poultry (all isolates identified as xx/2009 and xx/2015 in Supplementary Figure S2), in line with a niche specialization toward a human-associated environment. Moreover, an internal *O. intermedium* highly supported clade is exclusively composed of human isolates which includes two strains isolated from gastric disorder in association with *H. pylori* (Kulkarni et al., 2013, 2014) and the type strain of clinical origin (see Supplementary Figure S2). Whether this clade represents an ongoing pathogenic lineage should be carefully investigated with a more comprehensive epidemiological analysis. Similarly, more than 50% of the *O. anthropi* human isolates cluster in a well-supported clade that includes only human-related isolates (see Supplementary Figure S2). This cluster matches remarkably well the clonal complex CC4 observed by Romano et al. (2009) with 3 common strains, a complex exclusively composed of human clinical isolates in their study. Our results thus fully support the proposal of these authors that this lineage may represent a human-specialized opportunistic pathogen lineage among the environmental *O. anthropi* species.

## Conclusion

In summary, this study used a phylogenomic approach to investigate the taxonomic relationship of the family *Brucellaceae*. Our results confirmed the taxonomic position of the family *Brucellaceae* within the order of *Rhizobiales*, with the family *Bartonellaceae* containing the closest relatives. The family *Brucellaceae* includes, using this approach, the following current genera: *Pseudochrobactrum*, *Falsochrobactrum*, *Ochrobactrum*, and *Brucella*. The genus *Mycoplana*, described previously as belonging to the *Brucellaceae*, is actually positioned within the *Rhizobiaceae* and thus outside the *Brucellaceae*. Within the family *Brucellaceae*, while our approach confirmed the genus status of *Ochrobactrum* and *Pseudochrobactrum*, that of *Falsochrobactrum* was not totally resolved and that of *Brucella* appeared questionable. The various *Ochrobactrum* species clustered into two main clades. The first one includes *Brucella* spp. and the main opportunistic pathogenic *Ochrobactrum* species, such as *O. anthropi*, *O. intermedium*, *O. pseudintermedium*, and *O. haematophilum*. The second clade comprises the other *Ochrobactrum* species that seem currently not involved in opportunistic infections and are only exceptionally associated with humans. The robustness of the phylogenetic inferences we obtained here using two set of marker proteins and two sets of input genomes, compared to what was previously reported with single locus markers, emphasizes the help that genomic information can bring to characterize the taxonomic status of potential new species or genera. Nevertheless, according to our gANI analysis, the use of the 16S rRNA marker appears to be quite reliable to assign isolates to known species and should still be used for this purpose. Finally, the present work provides new elements on the question of the *Brucella* species delineation, and its status as a genus. Species definition has been problematic for numerous bacterial taxa because of a lack of a strong and universally accepted theoretical basis (Gevers et al., 2005). Our finding that all *Brucella* species can be considered as a single species according to gANI scores is actually also supported by the approach of the Genome Taxonomy Database (GTDB^2^) (Parks et al., 2018). This approach is an alternative to the official prokaryotic taxonomic nomenclature in which taxonomic ranks are defined according to a strict monophyly among groups and thresholds based on corrected genomic distances. Interestingly, under the GTDB framework, *Brucella* remains a genus, consisting of a single species as initially proposed by Verger et al. (1985). However, *Ochrobactrum* would be separated into three genera corresponding to the *O. haematophilum* clade, the *O. anthropi/tritici/intermedium* clade, and the *Ochrobactrum* clade 2 of the present study. The resulting GTDB classification remains, however, to be validated.

## Data Availability Statement

The datasets generated for this study can be found in the ncbi Biosample, SAMN12821270, SAMN12821271, SAMN12821272, SAMN12821273, SAMN12821274, SAMN12821275, SAMN1282 1276, SAMN12821277, SAMN12821278, SAMN12821279, SAMN12821280, SAMN12821281, SAMN12821282, SAMN 12821283, SAMN12821284, SAMN12821285, SAMN12821286, SAMN12821287, SAMN12821288, SAMN12821289, SAMN 12821290, SAMN12821291, SAMN12821292, SAMN12821293, SAMN12821294, SAMN12821295, SAMN12821296, SAMN 12821297, SAMN12821298, SAMN12821299, SAMN12821300, and SAMN12821301.

## Author Contributions

AC and MZ conceived and designed the study. SL performed *in silico* data analyses. SL, AC, and MZ analyzed the data and drafted the manuscript.

## Conflict of Interest

The authors declare that the research was conducted in the absence of any commercial or financial relationships that could be construed as a potential conflict of interest.

## Footnotes

1 ftp.wip.ncbi.nlm.nih.gov/genomes/genbank

2 http://gtdb.ecogenomic.org/
