# Supplementary figures and images for: Taxonomic Organization of the Family Brucellaceae Based on a Phylogenomic Approach
Source: Front Microbiol. 2020 Jan 30;10:3083. doi: 10.3389/fmicb.2019.03083 (PMC7002325; doi:10.3389/fmicb.2019.03083)

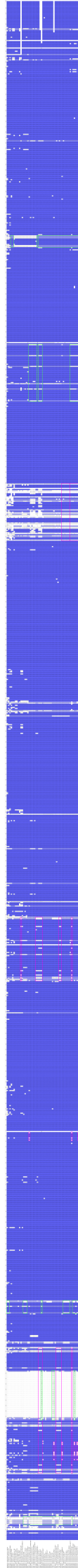

Supplement: Supplementary file 1 [file Image_1.pdf]

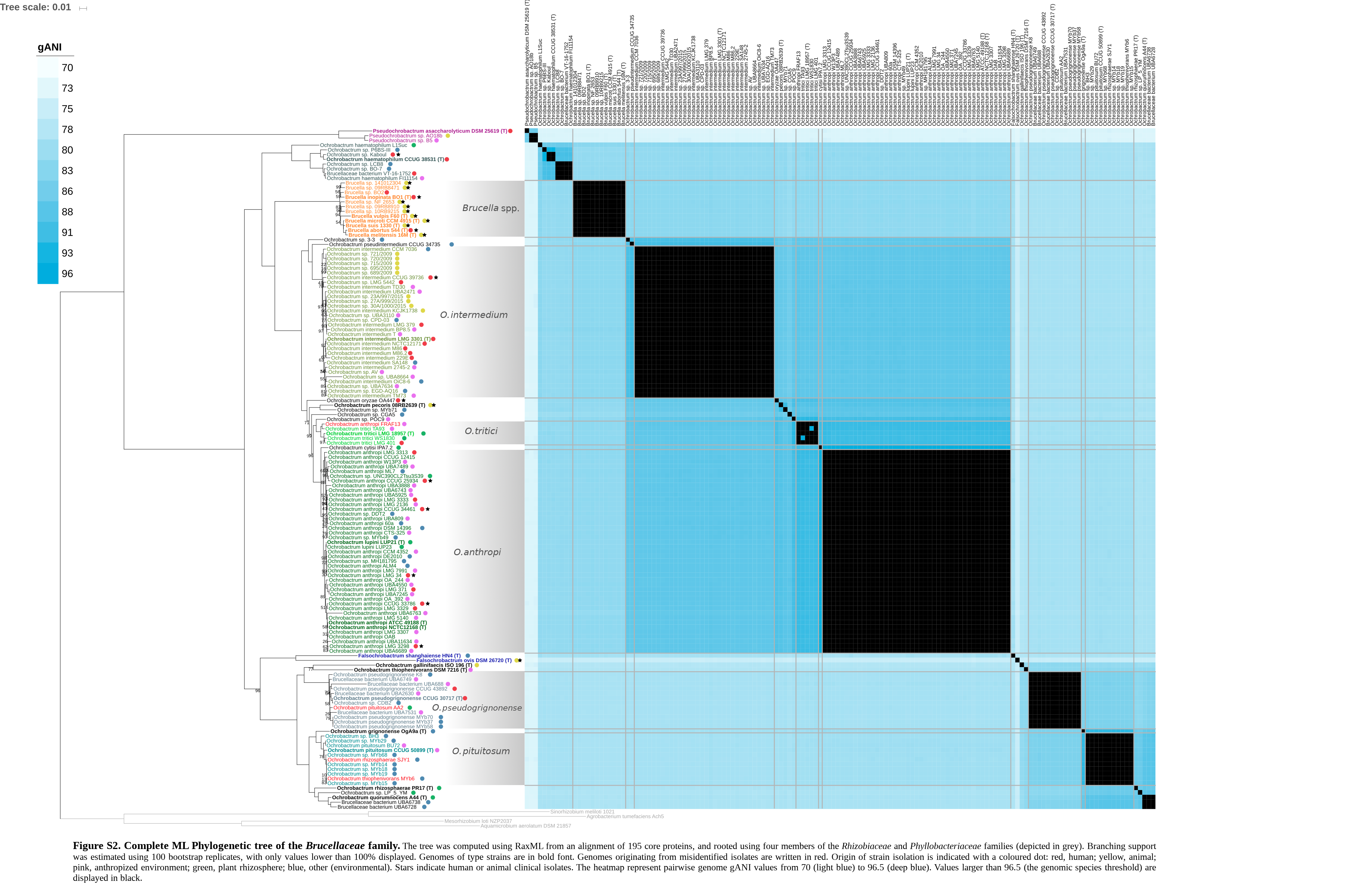

Supplement: Supplementary file 2 [file Image_2.tiff]

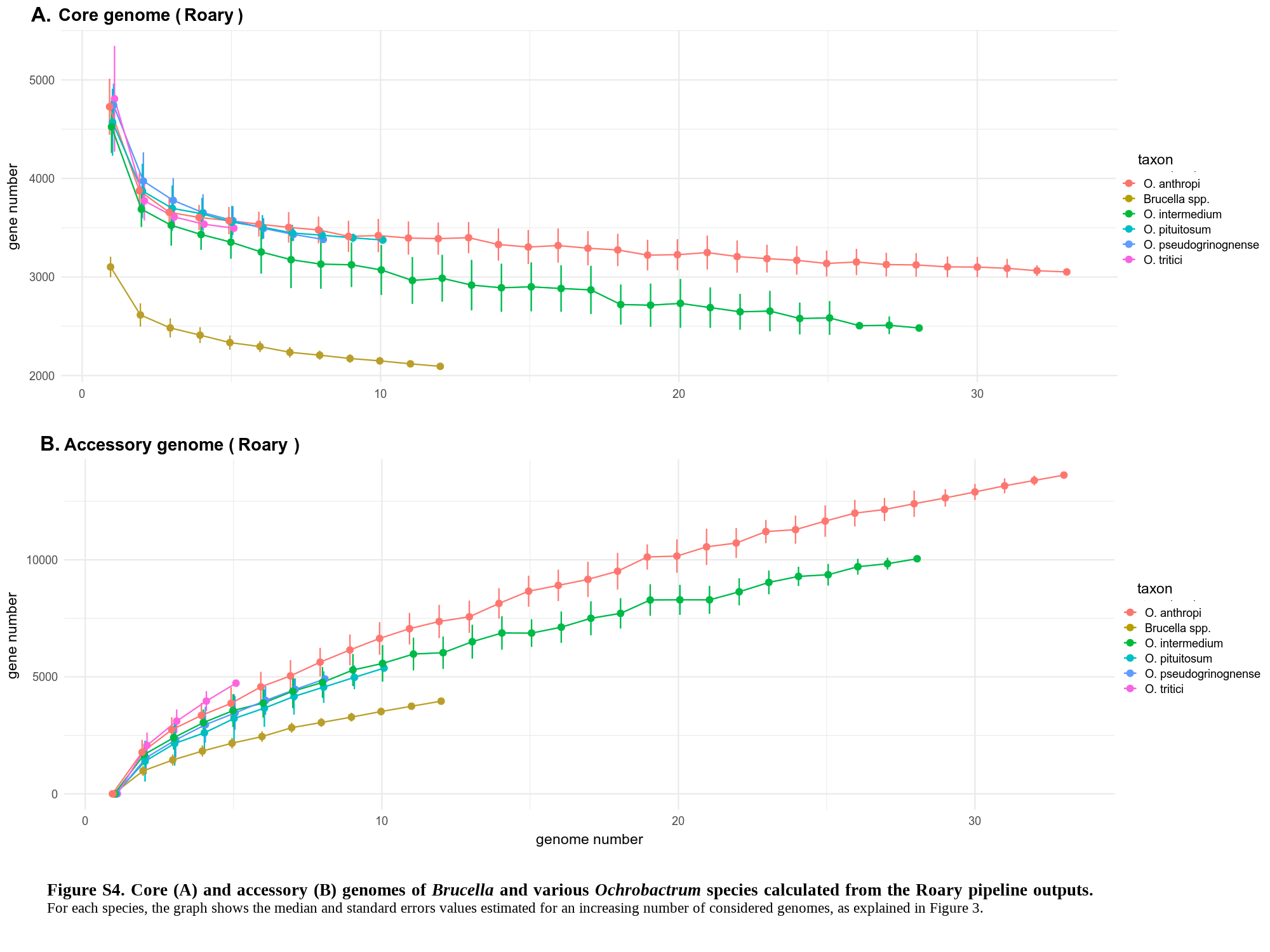

Supplement: Supplementary file 4 [file Image_4.tiff]
