# Supplementary material for: Taxonomic Organization of the Family Brucellaceae Based on a Phylogenomic Approach
Source: Front Microbiol. 2020 Jan 30;10:3083. doi: 10.3389/fmicb.2019.03083 (PMC7002325; doi:10.3389/fmicb.2019.03083)
Supplement: Supplementary file 3 [file Image_3.pdf]

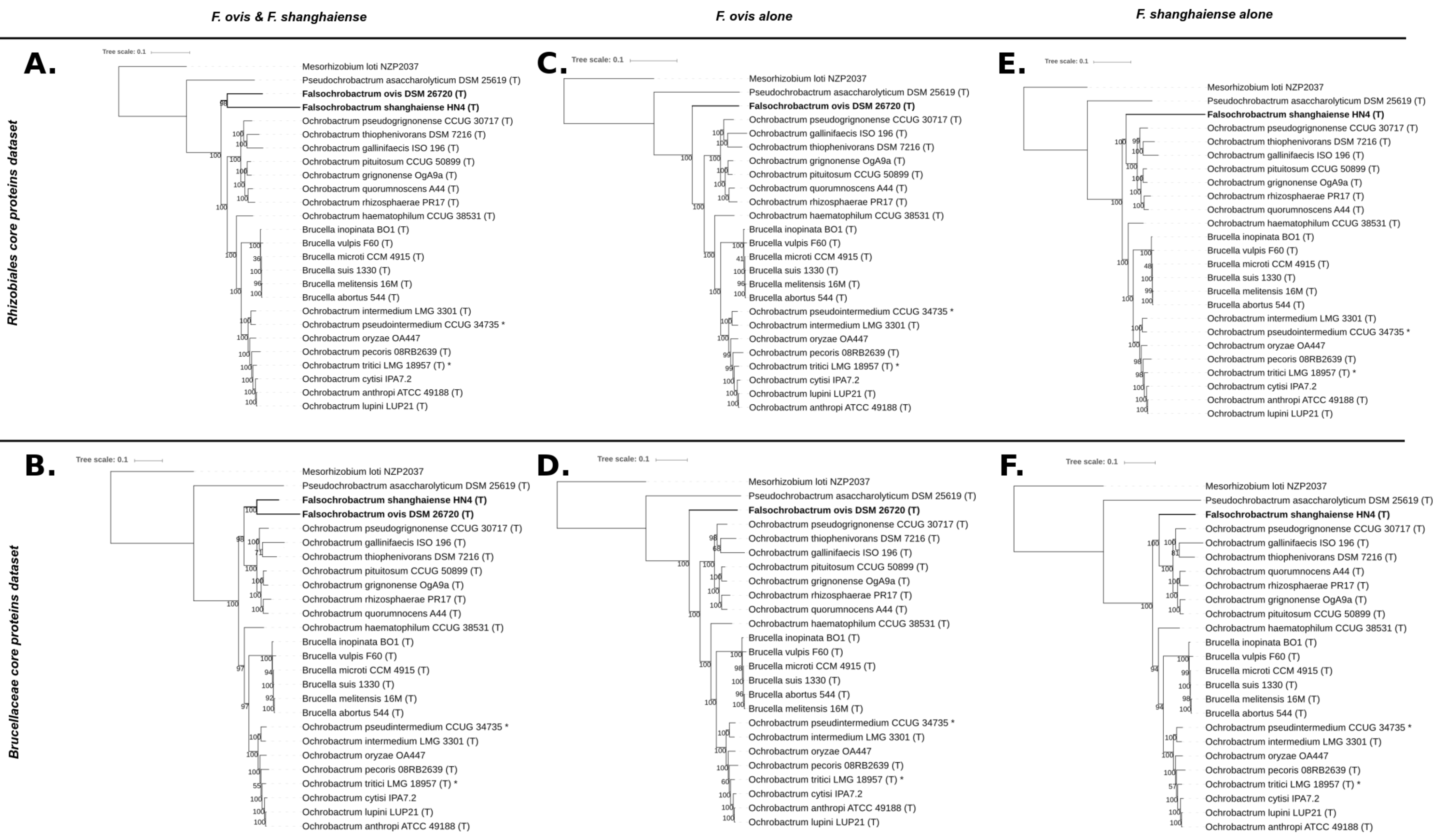

Figure S3. Position of *Falsochrobactrum* spp. among the family *Brucellaceae*. Various ML phylogenetic trees were computed using RaxML from the alignment of 145 and 195 core proteins (A,C,E and B,D,F, respectively), on datasets including representative *Brucellaceae* and both *Falsochrobactrum* (A,B), *F. ovis* only (C,D), or *F. shanghaiense* only (E,F). All trees were rooted using the *Mesorhizobium loti* genome. Branching support was estimated using 100 bootstrap replicates.
